# Preliminary characterization of the oral microbiota of Chinese adults with and without gingivitis

**DOI:** 10.1186/1472-6831-11-33

**Published:** 2011-12-12

**Authors:** Shi Huang, Fang Yang, Xiaowei Zeng, Jie Chen, Rui Li, Ting Wen, Chun Li, Wei Wei, Jiquan Liu, Lan Chen, Catherine Davis, Jian Xu

**Affiliations:** 1 BioEnergy Genome Center, Qingdao Institute of BioEnergy and BioProcess Technology, Chinese Academy of Sciences, Qingdao, Shandong 266101, China; 2 Procter & Gamble Innovation Center, Beijing 101312, China; 3 Procter & Gamble Winton Hill Business Center, Cincinnati, OH 45224 USA; 4 Department of Stomatology, Qingdao Municipal Hospital, Shandong, Qingdao 266011, China; 5 Department of Operative Dentistry and Endodontics, Guanghua School and Hospital of Stomatology, Sun Yat-sen University, Guangzhou 510055, China

**Keywords:** oral microbiota, gingivitis, saliva, plaque, pyrosequencing

## Abstract

**Background:**

Microbial communities inhabiting human mouth are associated with oral health and disease. Previous studies have indicated the general prevalence of adult gingivitis in China to be high. The aim of this study was to characterize in depth the oral microbiota of Chinese adults with or without gingivitis, by defining the microbial phylogenetic diversity and community-structure using highly paralleled pyrosequencing.

**Methods:**

Six non-smoking Chinese, three with and three without gingivitis (age range 21-39 years, 4 females and 2 males) were enrolled in the present cross-sectional study. Gingival parameters of inflammation and bleeding on probing were characterized by a clinician using the Mazza Gingival Index (MGI). Plaque (sampled separately from four different oral sites) and salivary samples were obtained from each subject. Sequences and relative abundance of the bacterial 16 S rDNA PCR-amplicons were determined via pyrosequencing that produced 400 bp-long reads. The sequence data were analyzed via a computational pipeline customized for human oral microbiome analyses. Furthermore, the relative abundances of selected microbial groups were validated using quantitative PCR.

**Results:**

The oral microbiomes from gingivitis and healthy subjects could be distinguished based on the distinct community structures of plaque microbiomes, but not the salivary microbiomes. Contributions of community members to community structure divergence were statistically accessed at the phylum, genus and species-like levels. Eight predominant taxa were found associated with gingivitis: TM7, *Leptotrichia*, *Selenomonas*, *Streptococcus*, *Veillonella*, *Prevotella*, *Lautropia*, and *Haemophilus*. Furthermore, 98 species-level OTUs were identified to be gingivitis-associated, which provided microbial features of gingivitis at a species resolution. Finally, for the two selected genera *Streptococcus *and *Fusobacterium*, Real-Time PCR based quantification of relative bacterial abundance validated the pyrosequencing-based results.

**Conclusions:**

This methods study suggests that oral samples from this patient population of gingivitis can be characterized via plaque microbiome by pyrosequencing the 16 S rDNA genes. Further studies that characterize serial samples from subjects (longitudinal study design) with a larger population size may provide insight into the temporal and ecological features of oral microbial communities in clinically-defined states of gingivitis.

## Background

Metagenomic techniques have recently revolutionized our understanding of the plethora of microbes that co-inhabit the human body, collectively known as the human microbiome. Various body sites (e.g. the skin, the gastrointestinal and vaginal tracts and the oral cavity) harbor distinct communities of microbes that vary among host individuals as well as among the ecological niches within each body site [1]. Interactions among resident microbiota and between the microbiota and the human host underlie human health and disease. Within the oral cavity, the tongue, soft and hard palates, buccal mucosa, supragingival and subgingival surfaces of the teeth and saliva may represent different ecological niches or habitats [2]. The composition and diversity of microbiota in these habitats may contribute to oral health [3-6] and oral diseases such as dental caries, periodontitis, and gingivitis [7,8].

Gingivitis is inflammation of the soft tissues of the gum surrounding the teeth. It is believed to result from the build-up of plaque [9] and the ensuing interactions between the plaque microbiota and host tissues [10,11]. These tissues become erythematous and bleed upon probing, but no apical migration of the junctional epithelium occurs. Previous studies of gingival plaque showed that as gingivitis develops, the microbial constituents of subgingival plaque shift from a population dominated by Gram-positive streptococci to one with elevated levels of Gram-negative anaerobes such as *Actinobacillus*, *Capnocytophaga, Campylobacter, Eikenella, Fusobactrium *and *Prevotella *[2,12,13]. However, these studies have been based on culture-based and molecular methods that target only a limited and partial number of culturable microbes, a bias that can be overcome by metagenomic approaches. During the last decade, high-throughput sequencing approaches based on 16 S rDNA amplicons have been used to survey the diversity of human oral microbiota in health and disease. Notably, these techniques revealed the microbial diversity within the healthy subgingival crevice exceeds far beyond that was characterized previously. Kroes *et al. *noted that less than a quarter (24%) of phylotypes identified with metagenomic techniques could be recovered by cultivation and that almost half of the subgingival phylotypes identified with a combination of molecular and culture-based techniques had not been characterized previously [14]. In fact, another study estimated that in human oral cavity approximately 68% of all bacterial taxa were still uncultivated [6].

Although molecular techniques have been used to compare subgingival plaques in healthy hosts and those with oral diseases such as periodontitis [15], few studies have investigated in depth the oral microbiota associated with gingivitis. There are several reasons. *First*, the depth and breadth of sampling for oral microbiota have been insufficient in general, and the optimal parameters not determined for that of gingivitis patients in particular. *Second*, regarding the selection of gingival sites for plaque sampling, it was still not clear whether or which of the different sites are clinically relevant (e.g., anterior teeth or posterior teeth? supragingival plaque or subgingival plaque?). Such ambiguity severely limits meaningful data analysis and comparisons across studies, and delays the translation of the findings clinically. *Third*, most oral microbial surveys that enumerated 16 S sequences of PCR-amplicons have ignored potential PCR artifacts [16-19]; as a result, a comprehensive and accurate organismal landscape of most oral microbiomes, particularly those related to diseases, remained largely elusive. All these factors have confounded the assessment of microbial factors associated with gingivitis.

Employing pyrosequencing of 16 S rDNA amplicons, this article elucidated the diversity and population structure of the oral microbiota, sampled respectively from five oral ecological niches from each of the three Chinese adults with gingivitis and three without the disease. Microbiota of supragingival plaque, subgingival plaque, and saliva were characterized to test whether and how the microbiomes from the various oral ecological niches distinguished healthy hosts and those with gingivitis. Our study pinpointed a number of organisms as potential biomarkers of gingivitis, and provided important insights for the sampling and analysis strategies for unraveling gingivitis-associated microbial risk factors in human populations.

## Results

### Study design

The six subjects were healthy, non-smoking adults in age ranging from 21 to 39 years (Table 1). Group assignment was based on the frequency of bleeding on probing (BOP). Three subjects were assigned to the healthy group (H) based on a BOP frequency ≤5 and three subjects to the unhealthy (gingivitis) group (U) based on a BOP frequency of ≥ 20. Group H consisted of three women and Group U included two men and one woman. Bleeding indices of the individual subjects were shown in Table 1.

**Table 1:** Metadata for the six subjects sampled in this study.

Group	Healthy (H)			Unhealthy (U)		
Subject ID	1	2	3	4	5	6
Gender	F	F	F	F	M	M
Age	22	23	21	39	25	25
Smoking	N	N	N	N	N	N
Chronic disease	N	N	N	N	N	N
BOP	5	1	1	24	37	25
MGI	1.1250	1.0357	1.0179	2.1346	2.6071	1.9286

Abbreviations: BOP - Bleeding on probing; MGI - Mazza Gingival Indexes

### Sequence datasets

Five samples (each from a different oral site; see **Methods**) from each individual were collected and analyzed. Barcoded 16 S-rDNA amplicon sequencing using 454 Titanium (average read length of 400 bp) yield a total of 494,988 raw reads, resulting in a dataset of 258,385 reads (after stringent quality assessment and control measures; **Methods**). The number of reads per sample ranged from 4,405 to nearly 13,562, with an average of 8,612 (Table 2).

**Table 2:** Estimates of species diversity for the samples.

Host ID	Site-specific sample	Sample ID	Raw reads	Reads analyzed	Unique sequence	Good's Coverage	OTUs at 3% difference	Ace	Chao 1
1	S	H1	18805	11580	3120	98.13%	687	902.15	944.54
	A-sup	H2	18448	9764	2307	98.56%	464	603.62	634.17
	A-sub	H3	15895	8035	2748	97.98%	597	740.87	775.64
	P-sup	H4	13974	7467	2142	96.69%	684	966.39	925.12
	P-sub	H5	14224	7730	2528	97.12%	702	932.10	923.01

2	S	H6	20982	11907	3264	98.35%	685	877.19	884.03
	A-sup	H7	19416	10935	2415	98.99%	379	491.50	490.02
	A-sub	H8	17295	9539	2060	98.66%	390	533.27	523.25
	P-sup	H9	22178	10085	2653	97.98%	631	846.61	842.29
	P-sub	H10	23105	10809	3160	98.06%	736	928.09	937.33

3	S	H11	19146	10970	3229	98.11%	612	847.56	878.51
	A-sup	H12	12515	5476	1933	97.11%	515	672.34	680.37
	A-sub	H13	13289	6581	1829	97.80%	466	608.60	631.71
	P-sup	H14	13105	6615	1896	97.57%	494	657.40	661.27
	P-sub	H15	12673	6456	1943	97.23%	539	728.34	757.23

4	S	U1	24258	13562	3929	98.50%	671	880.91	876.01
	A-sup	U2	18719	9591	2190	98.20%	495	700.72	713.79
	A-sub	U3	16282	7651	2636	97.56%	638	819.36	805.22
	P-sup	U4	20539	10035	3045	98.33%	617	766.07	749.34
	P-sub	U5	11515	5272	1963	96.47%	597	776.19	772.56

5	S	U6	20527	12890	3150	98.02%	722	1012.09	1001.18
	A-sup	U7	14307	7813	2436	97.82%	589	746.85	748.61
	A-sub	U8	14763	8078	2793	97.83%	621	785.67	781.26
	P-sup	U9	14787	7268	2213	97.14%	589	818.53	828.20
	P-sub	U10	16246	8916	2902	97.73%	656	858.40	934.10

6	S	U11	15739	8819	2569	97.74%	606	806.46	824.90
	A-sup	U12	12849	5407	2013	96.10%	570	819.64	810.82
	A-sub	U13	17990	8484	2844	97.15%	737	996.50	984.13
	P-sup	U14	9112	4405	1330	95.87%	475	678.74	671.08
	P-sub	U15	12305	6249	2189	96.13%	698	959.80	960.71

### Richness and diversity analysis based on Operational Taxonomic Units

**Figure 1:**
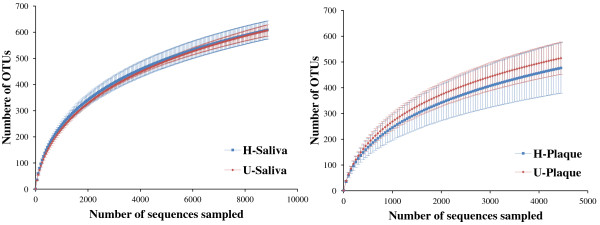
**Rarefaction curves for H and U groups at the sampled sites of saliva and plaque**. For both saliva plaque microbiomes, H and U Groups displayed similar phylogenetic diversity at 97% identity level (based on up to 4,000 sequences per sample).

Clustering the unique sequences into operational taxonomic units (OTUs) at a 3% genetic distance resulted in 464~737 different "species level" phylotypes per microbiome (Table 2). For all of the oral microbial communities analyzed, the number of OTU detected was very close to the total number of OTU estimated by Chao1 and ACE richness indicators. The average level of Good's coverage was over 97% in all samples, indicating that about three new phylotypes would be expected for every 100 additional sequenced reads. This level of coverage suggested that the 16 S rDNA sequences identified represent the majority of bacterial members present in saliva and plaque samples in the current study. The individual rarefaction curves showed a similar pattern of increasing diversity that has not yet reached saturation (Figure 1). Comparisons of the rarefaction curves in the healthy (H) and gingivitis (U) populations for the sampled sites of saliva and plaque showed that the two host-groups displayed similar richness of bacterial OTUs at 97% identity level.

### Comparisons of community structures

**Figure 2:**
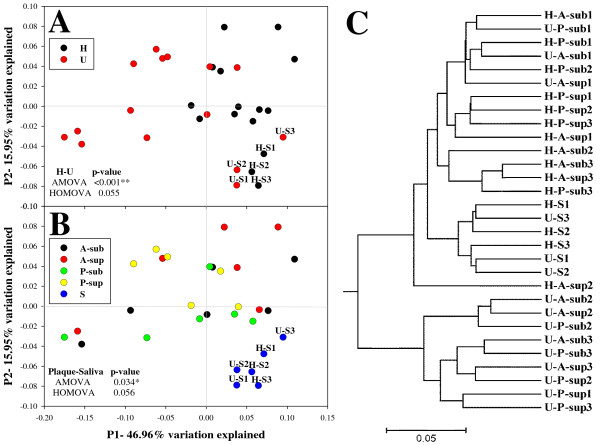
**Comparisons of bacterial community structures as measured by FastUniFrac**. Community structures from H and U Groups (**A**) or from the different sites (**B**) were interrogated using principal coordinate analysis (PCoA) and clustering analysis of the weighted UniFrac distance matrix. Each point corresponds to a microbial community, with color indicating its category. Percentages of variation explained by the plotted principal coordinates were indicated on the axes.

**Figure 3:**
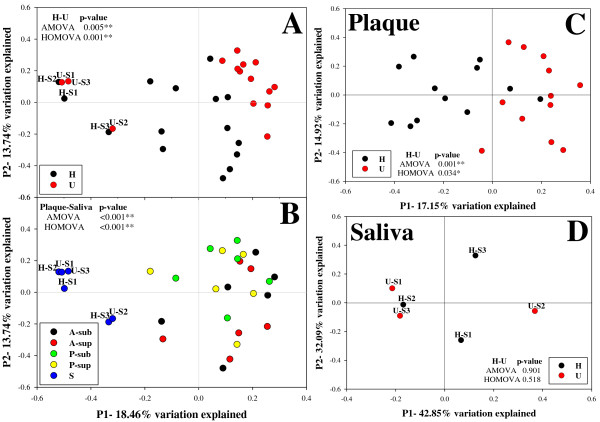
**ThetaYC-based analysis of bacterial community structures**. Community structures from H and U Groups (**A**) or from the different sites (**B**) were interrogated using principal coordinate analysis (PCoA) of thetaYC distance matrix. Each point corresponds to a microbial community, with color indicating its category. Percentages of variation explained by the plotted principal coordinates were indicated on the axes.

To determine whether the microbiota from the saliva and plaque distinguished healthy hosts and those with gingivitis, multivariate analyzes were applied to compare the overall structure of microbiota from each oral ecological niche based on FastUnifrac-derived and thetaYC-based distance matrices. FastUniFrac [20] allows pairwise comparisons of the evolutionary distances between two microbial communities and measures similarities among microbial community-structures. In the PCoA analysis, under a weighted UniFrac scheme, segregation between H and U groups was observed (*p *< 0.001) when all samples or when only plaque samples were considered, but not when only saliva samples were considered (Figure 2A). Moreover, regardless of their host-group affiliation, saliva microbiota formed a distinct cluster from the plaque microbiota (*p *= 0.034) (Figure 2B, C). The thetaYC-based PCoA analysis showed consistent results (Figure 3A, B). When only plaque samples were considered, the structural segregation between "H" and "U" groups was more discriminating (*p *= 0.001) (Figure 3C) than when all samples were included (*p *= 0.005) (Figure 3A). Therefore, gingivitis- and healthy-gingival-microbiomes can be distinguished based on the distinct community structures of plaque microbiomes, but not the salivary microbiomes.

### Taxonomy-based characterization of oral microbiota

**Figure 4:**
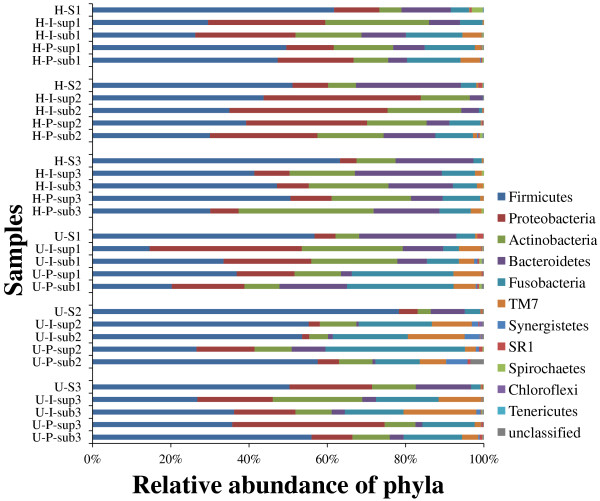
**Predominant phylotypes in each sample**. Over 90% of the diversity in each sample was contributed by the six phyla. However, variations were found in their relative abundances. See **Methods **for abbreviations.

**Figure 5:**
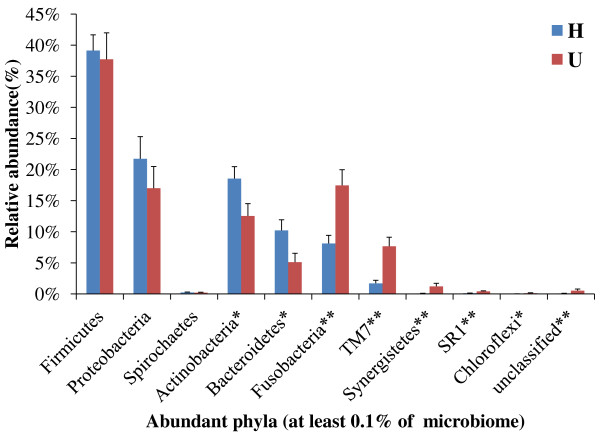
**Comparisons of bacterial taxonomic profiles of H and U groups at phylum level based on oral "CORE" database**. The relative abundance of each taxon between the H and U groups were compared using Metastats (*: 0.01 <*p <*0.05; **: *p <*0.01; mean ± s.e.m).

Bacterial phyla and genera were identified and quantified through taxonomic assignment against reference databases using MOTHUR, which reveal their relative abundance in all of the plaque and saliva microbiota (Figure 4; only phyla were shown). All sequences were found distributed in 11 bacterial phyla that include six predominant phyla commonly encountered in the oral cavity: Firmicutes, Proteobacteria, Bacteroidetes, Actinobacteria, Fusobacteria and TM7 [6,21]. The relative abundance of all plaque phyla detected in each of the two host groups suggested that significant differences for most of the phyla were found between hosts with or without gingivitis, except for Firmicutes, Proteobacteria and Spirochaetes (Figure 5). Among those gingivitis-associated phyla, Actinobacteria and Bacteroidetes were gingivitis-depleted while the remaining five phyla were gingivitis-enriched (Figure 5).

A total of 70 genera were identified in the oral microbiota from the five sampled sites (Additional file 1). The most frequently detected taxa at the genus level (the 12 most abundant genera that each represents at least 2% of oral microbiome) were *Streptococcus, Neisseria*, *Leptotrichia, Actinomyces, Prevotella *, *Veillonella, Rothia, Fusobacterium*, *Lautropia*, *Selenomonas, Haemophilus, Granulicatella*.

Statistically, 26 genera were found differentially distributed between gingivitis plaques (Group U) and healthy-gum plaques (Group H) (Table 3). Five genera (*Streptococcus, Veillonella, Prevotella *, *Lautropia *and *Haemophilus*) were significantly more abundant in healthy-gum plaque microbiota than those from gingivitis plaque, while the remaining 21 genera were found to be statistically less abundant.

**Table 3:** Bacterial genera differentially distributed between H and U groups based on the oral "CORE" database.

Genera	H		U		Metastats *p*-value	Metastats *q*-value
			
	mean abundance (%)	std.err	mean abundance (%)	std.err		
**Streptococcus**	**22.47%**	**1.91%**	**14.32%**	**1.98%**	**0.010989**	**0.0043**

**Veillonella**	**8.62%**	**1.90%**	**2.33%**	**0.77%**	**0.002997**	**0.0016**

**Prevotella**	**6.07%**	**1.75%**	**1.27%**	**0.44%**	**0.004995**	**0.0023**

**Lautropia**	**5.67%**	**2.00%**	**1.33%**	**0.31%**	**0.013986**	**0.0051**

**Haemophilus**	**4.15%**	**1.39%**	**0.58%**	**0.21%**	**0.000999**	**0.0008**

Leptotrichia	4.12%	0.91%	12.48%	2.44%	0.002997	0.001644

Selenomonas	1.73%	0.36%	6.71%	1.70%	0.002997	0.001644

Uncultured_Lachnospiraceae*	1.00%	0.27%	1.97%	0.25%	0.015984	0.0056366

Eubacterium	0.34%	0.08%	1.89%	0.34%	0.000999	0.000822

Cardiobacterium	0.60%	0.17%	1.19%	0.22%	0.038961	0.011658

Peptostreptococcus	0.19%	0.10%	1.42%	0.46%	0.002997	0.001644

Tannerella	0.20%	0.05%	1.36%	0.49%	0.000999	0.000822

Catonella	0.36%	0.10%	1.06%	0.25%	0.006993	0.003139

Synergistes	0.07%	0.03%	1.22%	0.49%	0.001998	0.001409

Filifactor	0.05%	0.04%	0.96%	0.28%	0.000999	0.000822

Peptococcus	0.12%	0.04%	0.49%	0.11%	0.004995	0.002349

Solobacterium	0.11%	0.03%	0.46%	0.22%	0.048951	0.01381

SR1	0.11%	0.04%	0.42%	0.08%	0.000999	0.000822

Syntrophomonas	0.00%	0.00%	0.17%	0.07%	0.000999	0.000822

Johnsonella	0.01%	0.01%	0.14%	0.05%	0.000999	0.000822

Choroflexus	0.01%	0.01%	0.12%	0.07%	0.030969	0.010172

Olsenella	0.01%	0.00%	0.05%	0.02%	0.041958	0.012185

Propionivibrio	0.00%	0.00%	0.05%	0.02%	0.032967	0.010172

Peptoniphilus	0.00%	0.00%	0.04%	0.02%	0.000999	0.000822

Desulfomicrobium	0.00%	0.00%	0.02%	0.01%	0.000999	0.000822

Pseudoramibacter	0.00%	0.00%	0.02%	0.01%	0.000999	0.000822

At genus level, we identified 26 gingivitis-associated taxa in plaque microbiota, which included five gingivitis-depleted taxa (bold) and 21 gingivitis-enriched taxa (without bold); *, not a well-defined genus.

### OTU-level comparisons of oral microbiota

As the above comparisons on the relative abundance of microbial taxa were derived from only those reads with confidence value above 0.8 (**Methods**), essentially all those reads without any reliable phylogenetic assignments (0.33~27.86% of the total reads in each sample) had been masked. Therefore, we further compared, between the two host-groups, the relative abundance of all species-level OTUs in plaque microbiota, independently of their phylogenetic identity assignments. In total, 98 OTUs (accounted for 5.38% of all OTUs) were found differentially distributed at the significance cutoff of 0.05 (both *p*-value and *q*-value of Metastats) in not only each of the four plaque sites but in all the plaque sites. Interestingly, among those 98 OTUs, 36 of them were gingivitis-depleted and the remaining 62 were gingivitis-enriched (Additional file 2). Consensus taxonomy of the OTUs was interrogated by MOTHUR based on oral "CORE" 16 S rDNA Gene Database (**Methods**). Twenty-four out of the 36 gingivitis-deplete OTUs while 57 out of the 62 gingivitis-enriched OTUs were supported by the taxonomy-assignment based results. Thus results from the two methodologies are largely consistent. These gingivitis-depleted or gingivitis-enriched OTUs represent a novel set of potential organismal markers for evaluation and prognosis of gingivitis, although their validity and significance remain to be further tested in larger human populations.

### Correlation of microbial quantifications between pyrosequencing and quantitative PCR (qPCR)

The abundances, in gene copies per ng of DNA, of two genera (*Streptococcus *and *Fusobacterium*) in all of the plaque and saliva samples were determined with qPCR. The relative abundance of these two genera as determined by pyrosequencing showed a positive correlation to the qPCR-based gene copies per ng of DNA (*Streptococcus*: *r *= 0.554; *p *< 0.002; *Fusobacterium*: *r *= 0.813; *p *< 0.001) (Additional file 3).

## Discussion

This study employed highly paralleled pyrosequencing of 16 S rDNA to assess and compare the diversity and population structure of microbiota associated with gingivitis in Chinese adults. The microbial diversity in plaque and saliva estimated in our study, 464~737 OTUs (97% identity cutoff) in each sample, was similar to that reported by Zaura et al (saliva; [5]). The Zaura study employed a stringent and conservative read-trimming strategy, where only those reads present at least five times in one sample were taken into analysis. In our analysis, stringent quality-based read-trimming suggested by MOTHUR was performed, requiring average quality score of over 35 in a 50 bp moving window along the whole read (**Methods**). This conservative selection criterion http://www.mothur.org/wiki/ significantly reduced the number of OTUs from the estimates based on alternative read-trimming criteria such as requiring average base quality score > 25 (data not shown). Thus potential sequencing artifacts might inflate the observed bacterial diversity. Furthermore, the estimated Good's coverage showed that most of the bacterial phylotypes (> 97%) in the saliva and plaque of these healthy and gingivitis hosts were already identified in this study. The richness estimator of ACE and Chao1 also suggested that the majority of phylotypes (> 97%) were already represented by the sequences in our study.

Our study firstly aimed to assess whether communities from healthy and gingivitis-associated host populations differ in any specific site(s) of oral cavity. Both FastUnifrac-based and thetaYC-based analysis showed that saliva and plaque samples represented distinct microbiomes in the oral cavity. Regardless of disease status, salivary microbiota clustered distinctly from plaque microbiota, in each of the two distance matrixes tested. This likely reflected the different environmental conditions characterizing the two habitats. Plaque microbiota reside in biofilms on the tooth enamel surface and are affected by dietary composition, oral hygiene practices [22], microbial interactions within the biofilm [8] and interactions between microbes and host epithelial cells [10,11,23]. In contrast, the salivary habitat was shaped by food intake flux, transient microbiota, mucins, serous exudate, sloughed epithelial cells, etc [3,4,24,25]. Interestingly, a survey of global diversity of the human salivary microbiome in ten individuals from each of twelve geographic locations worldwide (including China) reported a high diversity within and between host individuals but little geographic structure in the saliva microbiomes [26].

Secondly, members of the bacterial communities were identified. Furthermore, their contributions to the structural segregation of plaque microbiota between the two host populations were evaluated. When plaque microbiota were considered at the level of phylum, Fusobacteria and TM7, two of the predominant phyla, were more abundant in microbiota associated with gingivitis, while Actinobacteria and Bacteroidetes were less abundant in gingivitis-associated microbiomes. At the level of genus, several genera such as *Leptotrichia *and *Selenomonas *were more abundant in gingivitis plaque (21 such genera in total; Table 3), whereas only five genera, *Streptococcus*, *Veillonella*, *Prevotella*, *Lautropia *and *Haemophilus*, were less abundant. At species level, phylogeny-assignment independent comparison of relative abundances of OTUs between the healthy and gingivitis hosts was performed for not only each of the four plaque sites but also all of the plaque sites. Consistent with the above findings, 98 gingivitis-associated (both gingivitis-enriched and gingivitis-depleted) OTUs were pinpointed and found distributed in all sampled sites of plaque. Moreover, 58 OTUs affiliated to the genera of *Leptotrichia *(16), *Selenomonas *(12), *Streptococcus *(7), *Veillonella *(6), *Prevotella *(6), *Lautropia *(2), *Haemophilus *(3) and the candidate division TM7 (6) were found to be associated with gingivitis.

Notably, several members of these gingivitis-associated taxa were known to play a role in both oral health and disease. The gingivitis-enriched genus *Leptotrichia*, of the Fusobacteria phylum and Fusobacteriaceae family, were Gram-negative non-sporing-forming, anaerobic, saccharolytic rods. They were among the normal microbiota in the healthy oral cavity [27] and intestine [28]. *Leptotrichia buccalis *was found in high prevalence in a study of the gingival crevice of Chinese patients with gingivitis and necrotizing ulcerative gingivitis [29]. In a model of experimentally induced gingivitis, children harbored three-fold greater proportions of *Leptotrichia *species and 2.3-fold greater proportions of *Selenomonas *species in subgingival plaque than adults treated in the same way [30]. Similarly, *Selenomonas *species are Gram negative anaerobes normally found in the buccal flora and associated with gingivitis [31,32] and periodontitis [33,34]. TM7 is a prominent bacterial phylum of over 200 phylotypes without cultivated representatives [35-37] and found in diverse environmental habitats (such as soil, freshwater, deep sea and hydrothermal vents). Members of the TM7 candidate division have been recently detected in various human body sites [6,38-40], and associated with the inflammatory pathogenesis of several diseases (periodontitis [41], vaginosis [42] and inflammatory bowel diseases [43]). The subgroup I025 was found in subgingival plaque primarily at diseased sites in periodontitis hosts, suggesting their potential role in the multifactorial process leading to periodontitis [41,44].

On the other hand, only five gingivitis-depleted genera were detected in the current study. *Streptococcus *is one of the most predominant genera in the human oral cavity. However, the "oral streptococci" are a highly heterogeneous group genetically [45]. Although most are opportunistic pathogens and have been linked with a variety of oral diseases [46-48], they are also considered commensals. Similar to our results, *Streptococcus sanguinis*, as well as *Lautropia mirabilis *and *Haemophilus parainfluenzae*, were recently associated with oral health [34,47]. The genus *Veillonella *represents a group of small, usually non-fermentative, strict anaerobic, Gram-negative cocci. They are found in the human oral cavity, the upper respiratory tract, small intestines and vagina. In a survey of subgingival plaque from 22 subjects, the majority of the subgingival *Veillonella *isolates were identified as *Veillonella parvula *[49]. *Prevotella *species are part of the normal human oral microbiota and are frequently isolated from oral infections such as periodontitis, dental caries and abscesses [15,50,51]. Black-pigmenting members of *Prevotella *were associated with oral diseases. Consistently, in this study, most *Prevotella *OTUs detected in healthy hosts belonged to non-pigmenting species except *Prevotella tannerae*. Once validated in larger surveys, these gingivitis-associated genera, including both gingivitis-enriched and depleted ones, may represent valuable biomarkers for gingivitis.

Pyrosequencing techniques, such as the one employed in this study, revealed vast phylogenetic diversity and variability of bacterial communities in the human oral ecosystem [20,52]. Characterization and quantification of community components allowed distinctions in community structure between healthy and diseased states to be explored for disease biomarkers and specific-microbe-targeted therapy. To our knowledge, this is the first organismal survey of gingivitis-associated microbiota using deep-sequencing techniques. Our preliminary findings formulate the basis for further studies that feature a longitudinal design and include a larger number of subjects. Ongoing technical improvements on phylogenetic-marker amplification (such as those targeting DNA-extraction bias, sequence chimerism caused by PCR, bias of PCR amplification, sequencing errors, unequal amplification of community members and the typically unknown variations in the rDNA-gene copy numbers among different residents [16-19,53]) and the increasing coverage of oral 16 S rDNA reference databases [54] should allow the dissection of gingivitis-associated microbial factors at even higher sensitivity and resolution.

## Conclusions

This study revealed that, first, microbiota from the four sampling sites for plaque (supragingival plaque and subgingival plaque from anterior teeth; supragingival plaque and subgingival plaque from posterior teeth; **Methods**) were similar to each other, yet were distinguishable from salivary microbiota. Second, community structures of plaque microbiota, but not saliva microbiota, can be used to distinguish gingivitis. Thus plaque should serve as the sampling site of choice in providing a microbial perspective for the disease. Third, a number of organisms were identified as gingivitis-associated (with several low-abundance gingivitis-specific genera detected; Table 3), which can serve as potential biomarkers. These results have important implications in the sampling and analysis strategies for surveying gingivitis-associated microbial risk factors. Our findings now enable further studies that examine the temporal development and epidemiology of microbial risk factors behind gingivitis. Furthermore, based on the gingivitis-associated microbiota identified in this study, an integrated organism- and gene-based survey of oral microbiomes at various clinically defined states should unravel the nature of microbial contribution to the development of gingivitis.

## Methods

### Study design

All oral samples were collected at the Hai Tai He Chang Clinical Research Center in Beijing with approval from P&G Beijing Technical Center (China) Institutional Review Board and in accordance with the World Medical Association Declaration of Helsinki (1996 amendment). ICH Guidelines for Good Clinical Practice (GCPs) were followed. Healthy subjects aged 18 years or older who had a minimum of 18 natural teeth were recruited from the Beijing area. Voluntary informed consent was provided. Individuals meeting the following criteria were excluded: current participation in another clinical study; use of antibiotic, anti-inflammatory or anticoagulant therapy within 30 days prior to examination; self-reported pregnancy or lactation; diabetes; a history of hepatitis or blood disorders such as hemophilia or leukocythemia; the presence of orthodontic appliances or removable partial dentures; significant oral pathology, such as advanced periodontal disease, hard or tissue tumors, or other conditions considered significant by the study director. Gingivitis was assessed using Mazza Gingival Index (MGI) as defined by Mazza in 1981 [55]. Specifically, probing was performed by a dentist on the mesiobuccal and the distolingual sites of each tooth, for a maximum of 56 sites. BOP (Bleeding on probing) frequency and mean MGI were recorded for each subject. The MGI is similar to the Loe and Silness Gingival index; both are validated indices for describing gingivitis [55]. The merit, however, of using MGI is that it combines measurements that address both the signs of inflammation as well as the degree of the severity of bleeding. Scores range from 0-5, with 0 assigned for normal appearing and healthy gingiva up to a score of 5 for spontaneous bleeding (without provocation). Five individuals with healthy gums and another five with extensive gingivitis were enrolled. Subjects were assigned to the healthy group (H) if there were less than ≤5 bleeding sites and to the unhealthy (gingivitis) group (U) when the frequency of bleeding sites was ≥ 20. No randomization among groups was performed. Two subjects from each group did not return for follow-up examinations and were excluded from further analyses. In the end, a total of six subjects (three in each group) completed the full study.

### Sampling procedure

Samples of dental plaque and saliva were collected in the morning, 12 hours after evening tooth brushing. No oral hygiene or intakes of food and drink were allowed in the morning before sampling. Five samples were collected from each subject: supragingival dental plaque from anterior teeth (3~4 upper incisors), denoted A-sup; subgingival plaque [2 mm below gingival margin] from the same teeth, denoted A-sub; supragingival plaque from posterior teeth (2~3 upper molars), denoted P-sup; subgingival plaque from the same teeth, denoted P-sub; and saliva, denoted S. In the healthy group, plaque samples were collected from non-bleeding sites; in the unhealthy (gingivitis) group, incisor plaques were collected from non-bleeding sites and molar plaque were collected from bleeding sites. For unhealthy subjects, there are both non-bleeding incisor sites and bleeding molar sites for collecting plaque. We have considered the possibility that samples from the bleeding sites might not represent a complete picture of the microbiome of unhealthy gum. Therefore, plaque samples from both non-bleeding and bleeding sites were collected in our study.

Dental plaque samples were collected with sterile Gracey curettes and then removed from the curettes with a cotton-tipped swab. The tip of the swab was then placed into 0.4 mL lysis buffer (20 mM pH 8.0 Tris, 2 mM EDTA, 1.2% Triton X-100) and vortexed for 30 s. To collect salivary samples, subjects rinsed the mouth with 10 mL 0.9% saline buffer for 1 min and expectorated into a 50 ml tube. All samples were stored under -70°C before total genomic DNA extraction.

### DNA extraction and PCR amplification

Bacterial pellets collected from dental plaque and saliva were suspended in lysis buffer with lysozyme (20 mg/ml) and incubated with proteinase K. Bacterial DNA was extracted using QIAamp DNA Mini Kit (QIAGEN, Hilden, Germany) following the manufacturer's instructions. PCR amplicon libraries of the small subunit ribosomal (16 S) RNA gene V1-V3 hypervariable region (*Escherichia coli *positions 5-534) were generated for each individual sample. PCR were performed using the forward primer (NNNNNNN-TGGAGAGTTTGATCCTGGCTCAG) and reverse primer (NNNNNNN-TACCGCGGCTGCTGGCAC). Unique heptad-nucleotide sequences (seven bases) were synthesized at 5' end of each pair of primers as barcodes, which helped to assign sequences to different samples.

The amplification mix contained 12.5 ul of Gotaq Hotstart polymerase 2 × mix (Promega, USA), a 1 ul of each primer (5 pM), 1 ul genomic DNA (0.1-10 ng μl^-1^) and 9.5 ul H_2_O in a total volume of 25 μl. Cycling conditions were an initial denaturation at 95°C for 2 min, 25 cycles at 94°C for 30 s, at 56°C for 25 s, and at 72°C for 25 s, followed by a final 5 minute extension at 72°C. Samples were processed via separate PCR reactions (ABI StepOnePlus™ Real-Time PCR Systems) and then pooled. Each sample was amplified using one specific barcoded primer. To assess quality, the PCR product for each sample was subjected to electrophoresis (1.2% agarose, 5 V cm^-1^, for 40 min). Gels were stained with a buffer containing SYBR Gold Nucleic Acid Gel Stain (Invitrogen, USA); DNA fragments of approximately 500 bp were excised from the gel and further purified using Qiagen MiniElute kit. Concentrations of DNA in purified PCR products were further analyzed with PicoGreen (Invitrogen, USA). The amplicons were pooled into a single tube in equimolar ratios. Pyrosequencing of the 16 S PCR-amplicons was carried out on Genome Sequencer FLX Titanium (Roche, USA) where, on average, 400 bp-long reads were produced.

### Sequence processing

The sequences generated from pyrosequencing were mainly analyzed with MOTHUR [56] for preprocessing, identification of operational taxonomic units (OTU), taxonomic assignment and community-structure comparisons. To minimize the effects of random sequencing errors and avoid overestimates of the phylogenetic diversity [57], relatively stringent quality-based trimming of the reads was performed. First, the 454-reads were removed if they were < 150 bp, had an average quality score < 35 in each 50-bp window rolling along the whole read, had an ambiguous base call (N), had any homopolymers of more than eight bases or did not contain the primer sequence; reads were then sorted by the tag sequences. To reduce sequencing noise from pyrosequencing data, we performed the pre-clustering step [58] with the "pre.cluster" script in MOTHUR [56]. We also removed chimeric sequences detected by UCHIME [59].

### Operational Taxonomical Units (OTU) assignment and taxonomic classification

The trimmed reads were assigned to clusters using UCLUST http://www.drive5.com/uclust/. An in-house perl script was used to convert UCLUST output into a format recognized by MOTHUR [56]http://www.mothur.org/ for further analysis. Reads were assigned to OTUs (species-level). Calculation of coverage percentage (Good), species richness estimators (ACE and Chao1) and rarefaction analysis were performed using MOTHUR [56]. The relative abundance of OTUs with 97%-identity between pair-wise samples or between groups of samples were compared.

For taxonomic assignments, we used the "classify.seqs" script in MOTHUR [56] to classify all trimmed reads based on Naive Bayesian method with oral "CORE" [21] taxonomy sequences as the reference database. The confidence score threshold was set to 0.8, such that those with bootstrap value below 0.8 were assigned as unclassified. Relevant abundances of the bacterial taxa at the phylum and genus level were calculated and compared.

The OTUs defined by a 3% distance level were phylogenetically classified using the "classify.otu" script in MOTHUR [56] with oral "CORE" database [21] and a taxonomy file describing the complete taxonomic information of each sequence in the database from domain to species (using a 51% confidence threshold). The consensus taxonomy for each OTU was obtained in this step.

### Comparisons of microbiota community structures

FastUnifrac-based community structure comparisons were performed [20]. In each sample, representative sequences from each OTU were chosen by selecting the longest sequence based on UCLUST. Each sequence was assigned to its closest relative in a phylogeny of the Greengenes core set [60] using BLAST's megablast protocol. The resulting sample ID mapping file and category mapping file were used as inputs to the unweighted and weighted FastUniFrac [20]. FastUniFrac allows pairwise comparisons of distances between two microbial communities in terms of the fraction of evolutionary history that separates the organisms. A distance (a measurement of the similarity in community structure between two microbiota) was computed for each pair of samples, both within a single population and across the two populations, to create a matrix of pairwise distances among all samples. These distances were then clustered to reduce dimensionality using PCoA [61]. PCoA is a multivariate statistical technique for finding the most important axes along which the samples vary. The principal coordinates (PC), in descending order, describe of the degree of variation that each of the axes in the new space explains. ThetaYC-based community structure comparisons were performed in parallel with MOTHUR [56]. ThetaYC ($D_{\theta_{YC}}=1-\frac{\sum_{i=1}^{S_{T}}a_{i}b_{i}}{\sum_{i=1}^{S_{T}}{\left(a_{i}-b_{i}\right)}^{2}+\sum_{i=1}^{S_{T}}a_{i}b_{i}}$) measures the dissimilarity between the structures of two communities [62], where *S_T _*is the total number of OTUs in communities A and B, *a_i _*is the relative abundance of OTU *i *in community A, *b_i _*is the relative abundance of OTU *i *in community B. A matrix of pairwise thetaYC-based distances among all samples was created for clustering and PCoA analysis.

### Validation of 16 S rDNA pyrosequencing data by qPCR

Quantitative PCR assays on selected species were performed to test the degree of correlation with 16 S rDNA pyrosequencing data. Two genera, *Streptococcus *and *Fusobacterium*, were frequently identified based on our taxonomy assignments of the reads. Therefore, we chose two pairs of primers and probes targeting these two genera to perform the quantitative assays for comparisons to the pyrosequencing data.

Genus-specific primers and TaqMan probes were used, as listed in Additional file 4. The oligonucleotide probes were labeled with the fluorescent dyes 6-carboxyfluorescein (FAM) at the 5' end and 6-carboxytetramethylrhodamine (TAMRA) at the 3' end. The specificities of the probe and primer sets for their target DNA were tested in duplicate with the TaqMan Universal PCR Master Mix. The optimized concentrations of the forward primer, the reverse primer, and the fluorogenic probe in the 20-μl reaction volume were selected to be 300 nM, 300 nM, and 200 nM, respectively. Amplification and detection by quantitative PCR were performed with the StepOnePlus™ Real-Time PCR Systems (Applied Biosystems, Foster City, CA, USA). For each quantitative PCR, 20 μl reaction mixtures containing 2-μl sample DNA, forward primer, reverse primer and TaqMan probe at the optimized concentrations (as described above) were placed in each well of a 96-well plate. Following the fast TaqMan thermocycling protocol, reaction conditions were set at 95°C for 20 seconds, followed by 40 cycles of 95°C for 1 second and 58°C for 20 seconds. Standard curves for each organism were plotted in duplicate for each primer-probe set using the *Ct *(the cycle number at which the threshold fluorescence was reached) values, which were obtained by amplifying successive 10-fold dilutions of a known concentration of bacterial DNA (*Streptococcus mutans *UA159 and *Fusobacterium nucleatum *subsp. nucleatum ATCC25586). Copy-numbers of the target genes (*tuf*-elongation factor Tu and 16 S rDNA) in standard samples were calculated by the genome sizes (*S. mutans *2.0 Mb and *F. nucleatum *2.2 Mb) and the copy-number per genome (one copy of *tuf *gene per cell of *S. mutans *and five copies of 16 S rDNA gene per cell of *F. nucleatum *[46,63]. One ng of *S. mutans *genomic DNA contains 4.63 × 10^5 ^copies of *tuf *gene while 1 ng *F. nucleatum *genome DNA contains 2.10 × 10^6 ^copies of 16 S rDNA gene. Based on these assumptions, the absolute copy number of a target gene was determined by referring *Ct *value to a standard cure measured on the same plate. The relative abundance of these bacteria in the 30 different oral specimens was normalized by the absolute quantity of DNA in the clinical samples.

### Statistical analyses

AMOVA (Analysis of Molecular Variance) were used to test whether two communities from H and U populations have the same centroid [64,65]. HOMOVA (Homogeneity of Molecular Variance) was employed to test whether the genetic diversity are similar between the communities from the H and U populations [65,66].

Relative abundance of OTUs and phylotypes were reported as mean ± SEM. Due to the small sample sizes of these oral-site-specific datasets, features that are differentially distributed (i.e. abundant) between populations were statistically detected using Metastats [67] via a web interface http://metastats.cbcb.umd.edu/detection.html. Frequency data of OTUs and phylotypes were converted into a Feature Frequency Matrix as the input to this analysis tool. To exclude the extremely sparsely-sampled features (OTUs/phylotypes), tests were applied only if the total number of observations of a feature (OTU/phylotype) in either population is greater than the total number of subjects in the population (i.e. the average number of observations across subjects for a given feature is greater than one). Metastats was performed using 1000 permutations to compute *p*-values in statistical tests. We set *p*-value threshold of significance as 0.05. To control the FDR (False Discovery Rate) within the entire set of tests, we only took those features whose *q*-values and *p*-values were both below 0.05 into considerations. Levels of confidence were denoted as: *: 0.01 <*p *< 0.05; **: *p *< 0.01.

In validating the pyrosequencing results, the relative abundance of selected genera (*Streptococcus *and *Fusobacterium*) as measured via 16 S-amplicon pyrosequencing was compared to the corresponding gene copy number as determined by qPCR. The Shapiro-Wilk statistics of the variables were statistically significant. The degrees of correlation between the two measured parameters were determined from the Spearman's nonparametric correlation coefficient, *r*. Statistical analyses were performed with R (version 2.13.1). All reported *p *values were two-sided, at a 95% confidence level.

## Competing interests

The authors declare that they have no competing interests. The co-authors Rui Li, Wen Ting, Chun Li, Wei Wei, Jiquan Liu, Catherine Davis, and Lan Chen contributed and/or conducted this study while being employed by The Procter & Gamble Company. These authors may own stock in the Company, but a direct financial gain or loss from the publication of this manuscript is not anticipated. Under a requirement of employment obligations of The Procter & Gamble Company to maintain confidentiality, the authors cannot declare the patent activity. Financial support from the Procter & Gamble Company, Cincinnati, OH, was used to design and conduct the study. Chinese Academy of Science finances the publication of this manuscript including the article-processing charge. There are no other financial or non-financial competing interests to declare.

## Authors' contributions

RL, JQL, SH, FY, and JX designed study; XWZ, JC, LC and CD contributed analytical tools, DNA isolation protocol and barcode PCR primers; FY, TW, CL, WW and RL performed study; SH, FY and JX analyzed data; SH, FY, CD, RL and JX wrote the paper. All authors read and approved the final manuscript.

## Pre-publication history

The pre-publication history for this paper can be accessed here:

http://www.biomedcentral.com/1472-6831/11/33/prepub

## Supplementary Material

Additional file 1 **The 70 genera identified in all samples and their distribution in each sample**. Click here for file

Additional file 2 **Gingivitis-associated OTUs detected in plaque microbiota**. Among the totally 98 OTUs, 36 of them were gingivitis-depleted (Blue) and the remaining 62 were gingivitis-enriched (Red). Click here for file

Additional file 3 **Pyrosequencing-based and qPCR based quantification of the relative abundance of community members**. The degree of correlation for each genus was examined using Spearman's nonparametric correlation analysis: *Streptococcus *(**A**; *r *= 0.554; *p *< 0.002) and *Fusobacterium *(**B**; *r *= 0.813; *p *< 0.001). Click here for file

Additional file 4 **Oligonucleotide primers and probes used for the qPCR**. Click here for file
