# Supplementary figures and images for: Preliminary characterization of the oral microbiota of Chinese adults with and without gingivitis
Source: BMC Oral Health. 2011 Dec 12;11:33. doi: 10.1186/1472-6831-11-33 (PMC3254127; doi:10.1186/1472-6831-11-33)

# Streptococcus

**A**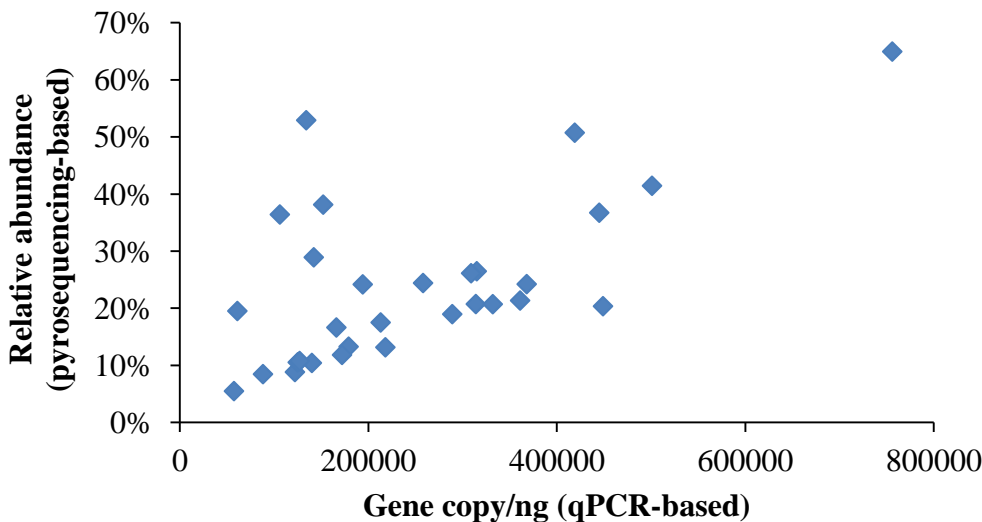

# Fusobacterium

**B**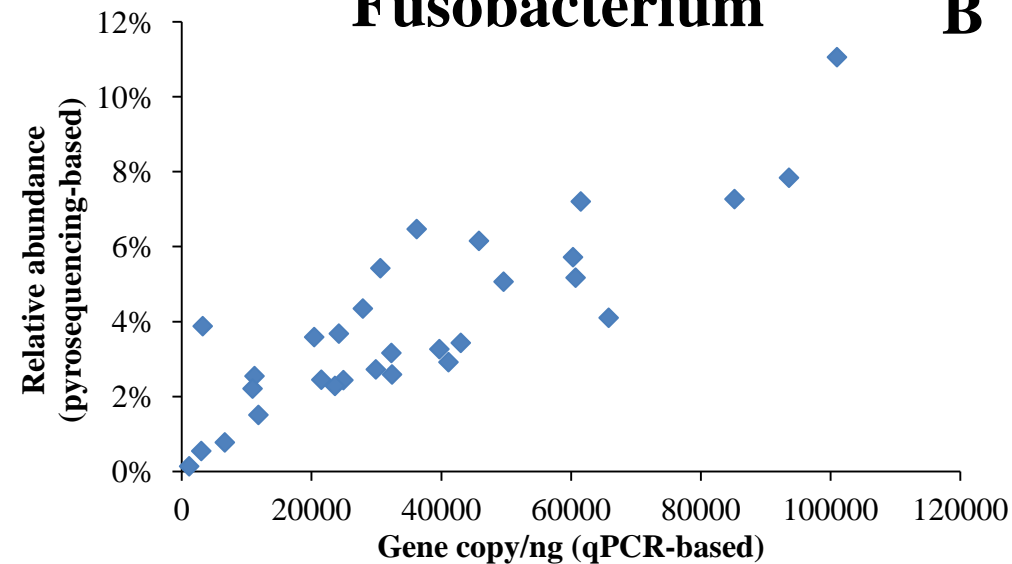

Supplement: Additional file 3 — Pyrosequencing-based and qPCR based quantification of the relative abundance of community members. The degree of correlation for each genus was examined using Spearman's nonparametric correlation analysis: Streptococcus (A; r = 0.554; p < 0.002) and Fusobacterium (B; r = 0.813; p < 0.001). [file 1472-6831-11-33-S3.PDF]
