# Supplementary material for: Preliminary characterization of the oral microbiota of Chinese adults with and without gingivitis
Source: BMC Oral Health. 2011 Dec 12;11:33. doi: 10.1186/1472-6831-11-33 (PMC3254127; doi:10.1186/1472-6831-11-33)
Supplement: Additional file 4 — Oligonucleotide primers and probes used for the qPCR. [file 1472-6831-11-33-S4.PDF]

Table S3

**Bacterium Targeted and primer or probe**	**Sequence**	**Source or reference**
Streptococcus		
Forward primer	5’-GTACAGTTGCTTCAGGACGTATC-3’	This study
Reverse primer	5’-ACGTTCGATTTCATCACGTTG-3’	
Probe	5’-FAM-GCAGTTGTTACTGGTGTTGAAATGTTCCGTA-TAMRA-3’	
Fusobacterium		
Forward primer	5’-AAGCGCGTCTAGGTGGTTATGT-3’	
Reverse primer	5’-TGTAGTTCCGCTTACCTCTCCAG-3’	1
Probe	5’-FAM-CAACGCAATACAGAGTTGAGCCCTGCATT-TAMRA-3’	

1.F. Martin,Nadkarni M.,N. Jacques,and N.Hunter.2002.Quantitative Microbiological Study of Human Carious Dentine by Culture and Real-Time PCR: Association of Anaerobes with Histopathological Changes in Chronic Pulpitis.J. Clin. Microbiol.40.5.1698–1704
